# Cerebral Collateral Circulation in Carotid Artery Disease

**DOI:** 10.2174/157340309789317887

**Published:** 2009-11

**Authors:** José R Romero, Aleksandra Pikula, Thanh N Nguyen, Yih Lin Nien, Alexander Norbash, Viken L Babikian

**Affiliations:** Departments of Neurology (JRR, AP, TNN, YLN, VLB), Neurosurgery (TNN), and Radiology (AN, TNN), Boston University Medical Center and Boston University School of Medicine, Boston, Massachusetts; the Framingham Heart Study (JRR, AP), Framingham, Massachusetts; and the Boston VAMC (VLB), Boston, Massachusetts, USA

**Keywords:** Carotid artery disease, cerebral collateral circulation, cerebral perfusion, stroke.

## Abstract

Carotid artery disease is common and increases the risk of stroke. However, there is wide variability on the severity of clinical manifestations of carotid disease, ranging from asymptomatic to fatal stroke. The collateral circulation has been recognized as an important aspect of cerebral circulation affecting the risk of stroke as well as other features of stroke presentation, such as stroke patterns in patients with carotid artery disease. The cerebral circulation attempts to maintain constant cerebral perfusion despite changes in systemic conditions, due to its ability to autoregulate blood flow. In case that one of the major cerebral arteries is compromised by occlusive disease, the cerebral collateral circulation plays an important role in preserving cerebral perfusion through enhanced recruitment of blood flow. With the advent of techniques that allow rapid evaluation of cerebral perfusion, the collateral circulation of the brain and its effectiveness may also be evaluated, allowing for prompt assessment of patients with acute stroke due to involvement of the carotid artery, and risk stratification of patients with carotid stenosis in chronic stages. Understanding the cerebral collateral circulation provides a basis for the future development of new diagnostic tools, risk stratification, predictive models and new therapeutic modalities. In the present review we discuss basic aspects of the cerebral collateral circulation, diagnostic methods to assess collateral circulation, and implications in occlusive carotid artery disease.

## INTRODUCTION

Atherosclerosis affecting the carotid artery is common in the general population and has been related to stroke [1], cognitive impairment [2] and dementia [3]. Symptomatic carotid artery stenosis has been implicated as the underlying mechanism in about 20% of ischemic strokes [4]. However, the risk of stroke in patients with carotid atherosclerosis is modified by several factors, including the clinical and demographic characteristics of the patients and the presence of collateral circulation. Thus, the clinical manifestations of occlusive carotid artery disease are highly variable; some patients may be diagnosed incidentally with asymptomatic carotid artery occlusion, while others present with devastating cerebral infarction. In addition, patients frequently have involvement of multiple intracranial and/or extracranial vessels, such as having disease in both carotid arteries. In one trial using angiography as a diagnostic method, bilateral carotid atherosclerosis was found in approximately 23% of patients [1]. In another study of patients undergoing carotid endarterectomy (CEA), occlusion of the contralateral carotid artery was found in 11.6% of patients [5]. Furthermore, the rates of stroke vary among these patients. Annual stroke rates in patients with carotid artery occlusion range from 0-5% in asymptomatic patients [6, 7] to 27% in symptomatic patients [8, 9]. In patients with bilateral occlusive carotid artery disease treated with CEA contralateral to a carotid occlusion, surprisingly low rates of stroke ipsilateral to the occluded artery have been reported (4% at 10 years) suggesting improved collateral flow in both the occluded and the operated sides [5].

One of the main factors explaining such variability in clinical presentation is the presence and effectiveness of collateral circulation [10]. The latter plays an essential role in preserving cerebral perfusion under physiological and pathological conditions where brain ischemia may occur. The cerebral collateral circulation can be recruited to preserve sufficient regional perfusion to brain tissue under different circumstances of ischemia and is recognized as an essential factor maintaining perfusion distal to the site of arterial occlusion [11]. In addition, the anatomical features of collateral circulation may determine patterns of stroke in occlusive carotid artery disease (i.e. borderzone vs. territorial) [12]. However, there are significant anatomical variations in the cerebral collateral channels among individuals, up to 50 % of the cases in the circle of Willis [13].

Conventional cerebral angiography remains the standard method to evaluate collateral circulation. There are specific advantages and disadvantages to cerebral angiography when compared with cross-sectional “global” studies of brain perfusion. The most conspicuous differentiating features are first, the focal physiologic interrogation of individually cannulated arteries as the individual angiographic acquisitions are performed to determine the regional supply attributed to each artery, and second, the rapid anatomic depiction of lobar and territorial flow without reliance on “reconstructed” image sets. Nevertheless other non-invasive methods have emerged including transcranial Doppler Ultrasound (TCD), magnetic resonance angiography (MRA) and computed tomography angiography (CTA). In recent years advances in imaging techniques have allowed for prompt evaluation of cerebral perfusion, and indirectly, the status of collateral circulation. Such methods are now widely available and provide valuable anatomical and functional information.

Evaluation of the cerebral collateral circulation may not alter the management for most patients with occlusive carotid artery disease. However, it may serve a prognostic role, and in a selected group of patients, it may provide additional information for risk stratification and therapeutic decisions.

In the present review we discuss anatomical and physiological considerations of the cerebral collateral circulation in the setting of carotid artery disease, diagnostic methods to evaluate the cerebral collateral circulation and clinical implications in acute and chronic situations.

## CEREBRAL COLLATERAL CIRCULATION

### Anatomy

Clinical and laboratory studies have provided information of the anatomical basis of the collateral circulation. The development of the cerebral collateral circulatory channels during embryonic stages parallels development of the nervous system. In the fetus, several arterial vascular channels interconnect the cerebral arteries during development [14]. After birth, some of these channels remain, constituting most of the anatomical basis of cerebral collateral circulation and forming direct anastomoses between neighbor arteries that belong to the same or different vascular territories. Variations in the cerebral collateral circulation between individuals are frequent and are important considerations for treatment, for instance in the case of endovascular therapies, or tumor resection. 

**Fig. (1). Cerebral Collateral Circulation.**
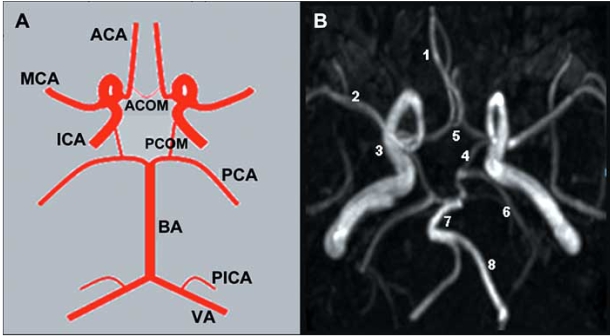
Panel A. Schematic representation of a complete circle of Willis. Panel B. Magnetic Resonance Angiography of the circle of Willis. 1= Anterior Cerebral Artery (ACA). 2= Middle Cerebral Artery (MCA). 3= Internal Carotid Artery (ICA). 4= Posterior Communicating Artery (PCOM). 5= Anterior Communicating Artery (ACOM). 6= Posterior Cerebral Artery (PCA). 7= Basilar Artery (BA). 8= Vertebral Artery (VA).

**Fig. (2). Common Variants of circle of Willis.**
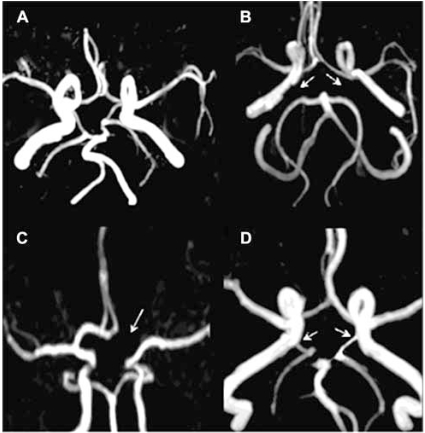
Panel A. Complete circle of Willis. Panel B. Absent Posterior communicating arteries bilaterally (arrows). Panel C. Absent left A1 segment of anterior cerebral artery (arrow). Panel D bilateral fetal origin of posterior cerebral arteries (arrows), i.e. arising from the internal carotid arteries.

The circle of Willis constitutes the main cerebral collateral network. Other collateral channels such as leptomeningeal collaterals and extracranial to intracranial collaterals, for example with retrograde flow in the ophthalmic artery, are considered secondary networks. New collateral channels can also develop after birth, but the process of new angiogenesis in the brain is not well understood. The collateral circulation of the brain is summarized in Table **1** and (Fig. **1**), and frequent anatomical variations are summarized in Table **2** and (Fig. **2**).

### Cerebral blood flow and collateral circulation

Brain blood flow is maintained relatively stable by the cerebral arterial autoregulation. Normal cerebral blood flow is approximately 50ml per 100grams of brain tissue per minute with differences between baseline and variable flow shown to gray and white matter. Cerebral blood flow is determined by the brain’s vascular resistance, perfusion pressure, and autoregulation. Cerebral perfusion pressure is determined by the difference between the mean arterial pressure and intracranial pressure (or jugular venous pressure). The cerebral circulation is capable of regulating blood flow within a wide range of changes in mean arterial pressure. In normal individuals, cerebral blood flow is maintained at a constant rate with mean arterial pressure fluctuating between approximately 50 and 150 mmHg. In addition, several mechanisms affect autoregulation, including blood pressure, metabolic, chemical and neurogenic factors.

### Recruitment of the Collateral Circulation

**Fig. (3). Recruitment of collateral circulation during carotid artery endarterectomy, Transcranial Doppler study.**
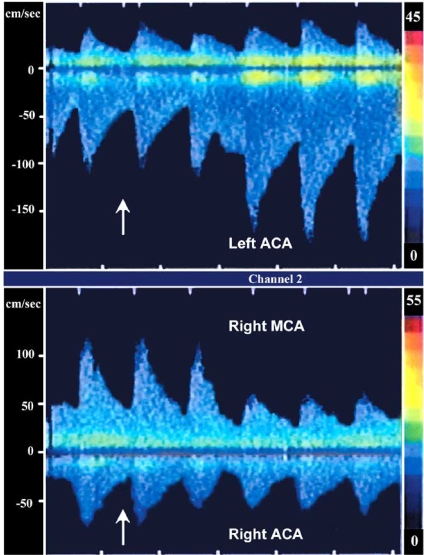
Inferior panel shows flow velocities in the proximal segment of the right middle cerebral artery (MCA) and right anterior cerebral artery (ACA). Two beats after clamping of the right internal carotid artery (arrow) there is a dramatic decrease in flow velocities in the ipsilateral middle cerebral artery and a less pronounced decrease in the ipsilateral anterior cerebral artery. The superior panel demonstrates increase in the flow velocity in the left anterior cerebral artery (negative deflection), contralateral to the clamped internal carotid artery, showing recruitment of collateral circulation.

The protective role of the collateral circulation depends on several factors including anatomical variations, systemic arterial pressure, age and the rate of development of occlusive disease. The circle of Willis constitutes the main network of collateral circulation and is immediately available to maintain perfusion in case of acute large artery occlusion. In addition to the circle of Willis, other collateral networks are also available but optimal functioning may develop over time. After occlusion of a large artery, the ensuing drop in perfusion pressure distally generates a pressure gradient between neighboring arterial fields, resulting in changes in flow direction and rate; collateral flow changes occur almost immediately, within 1-4 seconds [16], demonstrating that metabolic factors are less likely to account for this change. An example of rapid collateral recruitment through the circle of Willis can be seen during carotid endarterectomy. After clamping of the ipsilateral internal carotid artery, TCD monitoring shows an increase of the contralateral anterior cerebral artery flow velocities within two or three heart beats (Fig. **3**). A rapid drop in intraluminal pressure also results in relaxation of smooth muscle cells, vasodilation, and a drop of vascular resistance. This facilitates blood flow to ischemic tissue, as long as systolic blood pressure is maintained above 50 mm/Hg, otherwise the collateral circulation fails. This phenomenon is more prominent in small cortical arterioles 50 to 250micra in size [17].

In addition to the relatively rapid compensatory response, there is a slower response mediated by metabolic and other less well understood factors. In one experimental rat model of internal carotid artery occlusion, the posterior cerebral artery (PCA) enlarged by 39% after one week, and by 72% after three weeks; this was associated with the appearance of immunohistochemical markers of arteriogenesis [18]. In another experimental model, the basilar-carotid anastomoses enlarged over a period of 6 weeks [19]. In the case of gradual occlusion of the internal carotid artery, this response has been demonstrated to occur over several days following the occlusion. Angiogenesis or development of effective new conductive arterial vessels in the brain has not been conclusively proven in the human brain, with the exception of selected conditions such as moyamoya disease [20].

## DIAGNOSTIC METHODS

Different imaging modalities are available to evaluate the collateral circulation and brain perfusion, including TCD, Positron emission tomography (PET), Single-photon emission computed tomography (SPECT), CT-angiography (CTA), MR –angiography (MRA), perfusion CT (CTP), MR perfusion (MRP) and conventional cerebral angiography. Each of these techniques has advantages and limitations. Conventional cerebral angiography is considered the “gold standard” for evaluation of the anatomy of the collateral circulation, particularly leptomeningeal and external carotid artery supply, but has disadvantages including invasiveness, risk, and high cost.

**Fig. (4). Transcranial Doppler Ultrasound demonstrating patterns of collateral circulation.**
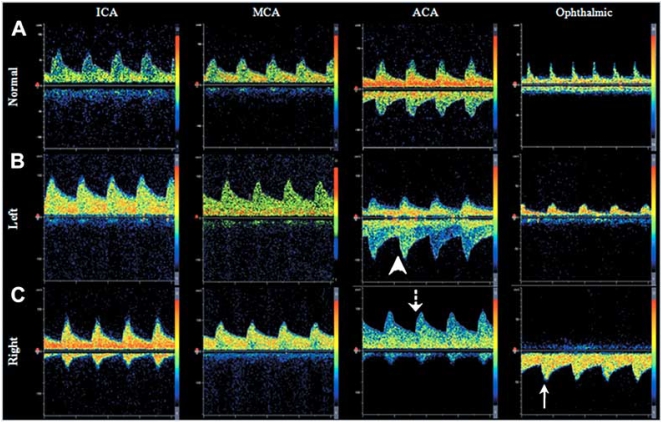
Panel A. Normal flow velocities and direction of flow in the internal (ICA), middle (MCA), and anterior cerebral arteries (ACA), and ophthalmic artery. Panels B and C. Example of recruitment of collateral circulation through the anterior cerebral artery (Panel B) and ophthalmic artery (Panel C) in a patient with right internal carotid occlusion below the level of the ophthalmic artery origin. Note that there is inversion of flow direction in the ACA (dotted arrow), compared to the normal side (arrow head). Panel C shows retrograde flow through the ophthalmic artery (solid arrow), demonstrating collateral circulation.

Transcranial Doppler Ultrasound is helpful in assessing cerebral flow patterns, vasomotor reactivity and identifying patients who are at risk for Stroke/TIA. TCD can determine flow patterns suggestive of collateral circulation in patients with carotid artery disease (Fig. **4**). For instance, presence of retrograde flow from an ophthalmic artery is detected in severe carotid stenosis (Fig. **4C**). Furthermore, TCD assists in detecting silent cerebral emboli during continuous monitoring, thus identifying a group of patients at higher risk of subsequent stroke/TIA [21]. TCD also is used for evaluation of vasomotor reactivity (VMR) to CO2, a surrogate marker of cerebrovascular reserve and autoregulation. A reduced vasoreactive response suggests impaired cerebral perfusion and poor collateral circulation [22, 23]. Previous studies have found an association of impaired VMR with higher risk of stroke/TIA in patients with carotid stenosis [23, 24], and also with increase in ischemic lesion volume in the borderzone ipsilateral to an internal carotid occlusion, supporting that brain areas extending between superficial and deep middle cerebral artery (MCA) branches are susceptible to hemodynamic compromise and impaired washout of microemboli [25].

Single-photon emission computed tomography also allows evaluation of regional cerebral perfusion and hemodynamic reserve in patients with carotid artery disease, by measuring regional VMR after administration of acetazolamide or CO2. This is especially helpful in assessing patients with multivessel or multiterritorial disease in whom determination is sought for identifying “greatest risk” territories for treatment. Patients with “misery perfusion” and reduced vasoreactivity are at risk for recurrent stroke [26, 27], and vasoreactivity spontaneously improves with unilateral internal carotid artery occlusion [28], indicating the recruitment of collateral circulation over time. In chronic carotid artery disease, SPECT is used at some centers to select appropriate candidates for intervention, by means of evaluating cerebrovascular reactivity [29].

**Fig. (5). Cerebral perfusion studies: Computed Tomography and Magnetic Resonance Imaging.**
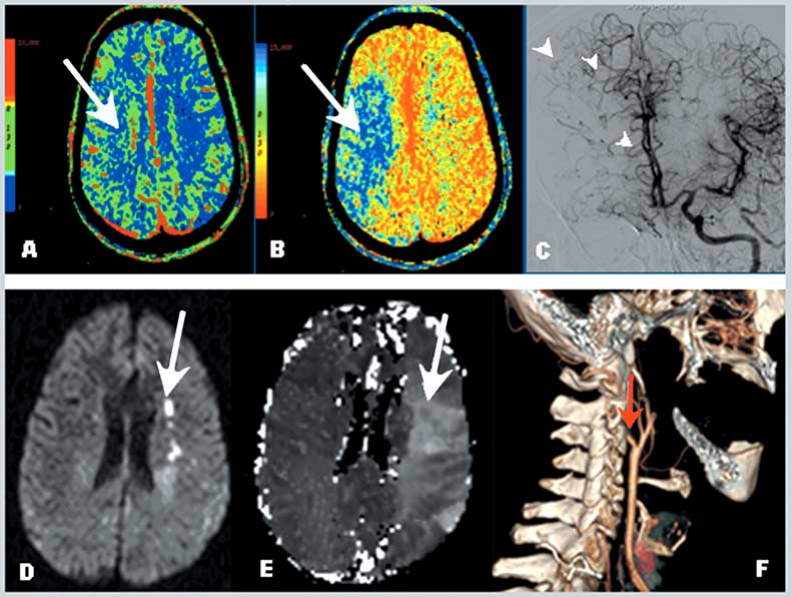
Panel A. Cerebral Blood Volume (CBV) demonstrates normal values. Panel B. Prolonged mean transit time (MTT) demonstrates hypoperfusion of the right hemisphere in the middle cerebral artery territory. Comparison of the CBV and MTT images reveals a mismatch, consistent with a region of ischemic penumbra. Panel C. Cerebral angiogram in the same patient showing collateral flow to the right anterior cerebral artery territory through the anterior communicating artery and leptomeningeal collaterals (arrow heads). Panels D, E and F. Example of diffusion weighted images (DWI) and perfusion weighted images (PWI) in a patient with carotid artery occlusion. Note small areas of restricted diffusion in Panel D (acute infarcts-bright signal) compared with a large area of decreased perfusion in Panel E (arrow), demonstrating a region of mismatch (ischemic penumbra). Panel F shows a three dimensional reconstruction of the carotid system based on CT angiography, demonstrating occlusion of the internal carotid artery at the origin (red arrow).

The CT-based methods, CTA and CTP, can be performed rapidly and are widely available to clinicians. Several quantitative maps are obtained to measure the cerebral hemodynamic parameters: cerebral blood flow (CBF), cerebral blood volume (CBV), mean transit time (MTT) and time-to-peak (TTP) [30]. In acute stroke, a brain region with prolonged MTT has decreased perfusion and impaired hemodynamics. Within this region, increased CBV identifies an area of preserved autoregulation and functional collaterals and tends to correspond to the penumbra or “tissue at risk”, while regions with decreased CBV correspond to the core infarcted tissue (Fig. **5**). As such, CTP can rapidly detect hypoperfused brain tissue in the acute stroke setting [31]. MTT demonstrates larger regions, which show prolonged contrast and blood transit times, and may be seen with chronic and stable conditions. MTT is the parameter that best agrees with PET oxygen extraction values in patients with chronic carotid occlusion [32].

MR Angiography (MRA) underestimates the presence of collaterals, but the use of contrast administration improves this limitation [33]. Similar to CT technology, MRI techniques with diffusion-weighted imaging (DWI) and perfusion-weighted imaging (PWI) (Fig. **5**) are helpful to identify the ischemic penumbra and to select appropriate patients for interventions in acute settings [34-36]. This method is in agreement with PET data when compared with DWI. Quantification of diffusion and perfusion disturbances by MRI can provide objective measure of an infarct core and surrounding penumbra [37]. MRP could potentially be employed for selection of patients with symptomatic occlusive carotid artery disease to assess the risk for subsequent clinical events if poor collateral flow is present.

Knowing that collateral circulation can sustain penumbra, Bang *et al.* evaluated the relation of perfusion parameters (DWI/PWI mismatch) and pretreatment angiographic collaterals to tissue fate in the immediate post- ischemic time frame. Patients with good collateral flow had less hypoperfused tissue and also less infarct growth within the penumbra zone than those with poor collaterals. These findings provide further support to the notion that poor collateral flow is an important determinant of brain tissue fate, particularly in the setting of poor/ partial recanalization after treatment and the presence of an extensive DWI/PWI mismatch on pretreatment MRI [38].

## CAROTID ARTERY DISEASE AND COLLATERAL CIRCULATION

Symptoms of cerebral ischemia may develop when there is brain embolism and/or collateral failure [17]. The latter also depends on systemic conditions. While conventional angiography is best suited to determine the anatomy of collateral circulation, the functional adequacy of the collaterals to preserve brain perfusion is better determined by a perfusion study. The effectiveness of the cerebral collateral circulation may vary in the setting of acute and chronic brain ischemia resulting from carotid artery stenosis/occlusion.

## CAROTID ARTERY STENOSIS AND COLLATERAL CIRCULATION: ACUTE SETTING

**Fig. (6). Cerebral Angiography in a patient with right internal carotid artery occlusion.**
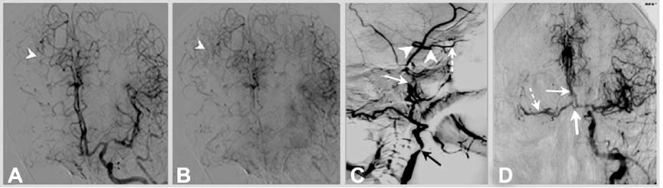
Panels A and B. Leptomeningeal pial collateral channels (arrow heads) to the right anterior and middle cerebral artery territory (Panel A early arterial phase, Panel B late arterial phase). Panel C. Retrograde filling of intracranial internal carotid artery (arrow heads) through the ophthalmic artery (dotted arrow). Stenosis of the extracranial internal carotid artery in the proximal segment at the origin (black arrow) and occlusion in the distal extracranial segment (white solid arrow) are seen. Panel D. Collateral flow to the right anterior (solid arrow) and middle cerebral (dotted arrow) arteries through the anterior communicating artery (large solid arrow), in same patient with right internal carotid artery occlusion.

Acute brain infarction due to carotid artery occlusion has been associated with poor outcome, while patients with robust collateral flow have better clinical outcomes [39]. Collaterals from the external carotid artery to the petrous segment of the internal carotid artery may be a helpful predictor for successful recanalization and an indicator of good prognosis [39]. Thus, identification of collateral channels by means of angiography has been suggested as one of the factors for patient selection for treatment with intrarterial thrombolysis followed by CEA in selected cases [40]. In patients undergoing intrarterial thrombolysis for acute stroke, those with good pial collaterals (Fig. **6**) have a lesser degree of infarct growth after the first symptoms of brain ischemia, smaller infarct volumes, and correspondingly better outcomes [41]. Moreover, collateral flow has a greater impact on ultimate infarct volume than time from symptom onset to treatment [41], and the pretreatment collateral status is independently associated with infarct growth [38]. Similar results were noted in the PROACT II trial; a collateral grading system was developed and the collateral scores predicted the extent and location of cerebral infarcts in patients with MCA occlusions [42].

Although available data are strongly indicative that good collateral flow is key in cerebral perfusion and in reducing infarct size, this knowledge has resulted in only limited therapeutic applications so far. Chief among measures intended to improve collateral flow is the management of blood pressure. In stroke units, higher blood pressure readings are accepted or pharmalogically increased temporarily in selected patients with collateral flow failure (Fig. **7**). However, these patients constitute a minority of patients with stroke.

The preceding considerations may have future practical applications. The use of imaging modalities evaluating cerebral perfusion has resulted in expansion of the therapeutic window for intervention in acute stroke. At present, use of combination therapies for the treatment of acute stroke, including strokes related to carotid artery occlusion, are the subject of research in randomized clinical trials using cerebral perfusion imaging techniques [43, 44]. Use of methods to enhance collateral flow in the setting of acute large artery occlusion include the use of devices selectively diverting blood flow to the cerebral circulation [45]. Evaluation of cerebral collateral circulation in clinical trials should be emphasized to aid in appropriate patient selection for acute interventional therapies.

## CAROTID ARTERY STENOSIS AND COLLATERAL CIRCULATION: SUBACUTE AND CHRONIC SETTING

The collateral circulation also has long been recognized as a factor modifying stroke risk in the presence of carotid stenosis [46]. A noncompetent circle of Willis is associated with border zone infarcts [47] (Fig. **7A**), whereas a complete circle of Willis in patients with carotid artery occlusion has been related to only minor or no neurological deficits [48]. The two principal mechanisms for stroke in patients with carotid artery occlusion or severe stenosis are hypoperfusion and embolism. In this setting, decreased cerebral perfusion and insufficient collateral circulation may exacerbate the impact of embolism, as microemboli are more likely to cause symptomatic brain ischemia due to impaired washout in hypoperfused areas [49, 50].

In symptomatic patients with severe carotid stenosis, the North American Symptomatic Carotid Endarterectomy Trial (NASCET) investigators evaluated the effect of angiographically demonstrated cerebral collateral circulation on the risk of stroke in patients treated medically and surgically (CEA). The presence of collateral circulation was found to increase with progressive degrees of carotid stenosis; next to nil collaterals were seen in patients with no stenosis, whereas collaterals were seen in more than 50% of patients with the most severe stenosis. In addition, the adjusted risk of hemispheric stroke was significantly lower in medically treated patients with collaterals compared to those without collaterals (13.3% versus 6.3%, for disabling or fatal stroke; 27.8% versus 11.3% for hemispheric stroke). The benefit of reduced risk of stroke was observed regardless of the degree of stenosis. In the group of patients treated surgically, a non significant reduction of the risk of any hemispheric stroke was seen in those with collateral circulation, both perioperatively (1.1 versus 4.9% in those with and without collateral circulation respectively) and at 2-year follow up (5.9 versus 8.4% in patients with and without collateral circulation respectively) [51]. Other studies have also recognized the value of collateral circulation as a prognostic factor in stroke due to large artery atherosclerosis [52].

Considerations of collateral circulation may affect therapeutic decisions for patients considered for extracranial to intracranial (EC-IC) bypass. This procedure has been used for cerebral blood flow augmentation in the setting of occlusive cerebrovascular disease. Although a prior EC-IC international trial failed to demonstrate a benefit for the procedure over medical therapy [53], there has been considerable progress in imaging techniques for evaluation of the hemodynamic status of the cerebral circulation allowing for better patient selection [54], as well as in the development of surgical techniques. Imaging techniques providing physiological and anatomical information of the cerebral circulation may allow selecting a subset of patients that may benefit from this procedure. Previous studies suggest that EC-IC bypass may be beneficial in selected patients with occlusive carotid artery disease [55, 56], and the procedure remains a treatment option for patients with Moyamoya disease [57]. At present a randomized clinical trial is evaluating the benefit of EC-IC bypass for patients selected using cerebral perfusion studies with recent symptomatic internal carotid artery occlusion [58].

The case for asymptomatic patients with carotid stenosis may be different. In contrast to the findings reported in NASCET, investigators from the Asymptomatic Carotid Atherosclerosis study (ACAS) reported a significant 2% increase in the absolute risk of stroke in patients with contralateral carotid occlusion treated with CEA compared to medical therapy (3.5 versus 5.5%) Although the collateral circulation was not assessed in this study and several factors are likely to account for the results, the authors argued that the results may be related to effectiveness of collateral circulation [59].

Frequent surgical practice challenges include the selection of patients for surgery, the decision regarding the side to operate in patients with bilateral ICA stenosis, and technical considerations such as the use of a shunt. This is especially true in cases where the patient’s disposition is not clearly apparent based on prior clinical trials. Evaluation of cerebral hemodynamic factors using perfusion studies and studies of cerebrovascular reserve may aid in decision making. Patients with decreased cerebrovascular reserve are at a greater risk of stroke and have greater dependence on collaterals. Thus, evaluation of the cerebral collateral circulation may help stratify the risk in patients with severe bilateral carotid artery disease. Patients with poor collateral flow and decreased cerebrovascular reserve also are at a greater risk for stroke and thus may benefit from revascularization, whereas patients with adequate collateral circulation, preserved cerebral perfusion and vascular reserve, particularly if asymptomatic, may be better served with medical treatment. These variables can tilt the decision for revascularization in the individual patients, but general recommendations cannot be made based on the present available data.

## CONCLUSION

The cerebral collateral circulation plays an important role preserving brain tissue perfusion in physiological and in pathological conditions where ischemia may develop. The predictive role of evaluation of the collateral circulation in patients with carotid artery disease has been consistently demonstrated in previous studies. The presence of good collateral circulation is a favorable prognostic factor and its absence is considered an unfavorable prognostic indicator both in acute and chronic settings for patients with symptomatic carotid artery disease. Evaluation of the cerebral collateral circulation in conjunction with cerebral perfusion and the clinical characteristics of patients with occlusive carotid artery disease may provide a more comprehensive view of the cerebrovascular condition. Although at present the clinical applications of information regarding collateral circulation for patients with carotid artery disease are not entirely clear, such assessment may have implications for therapeutic decisions in the acute setting of carotid artery occlusion and stroke. Future research developments in imaging studies will likely advance our understanding of the role of the cerebral collateral circulation in patients with occlusive carotid artery disease, both in acute and chronic situations.

## Figures and Tables

**Fig. (7). Example of patient with carotid artery occlusion and clinical fluctuations related to hemodynamic factors; evaluation of collateral circulation and cerebral perfusion.**
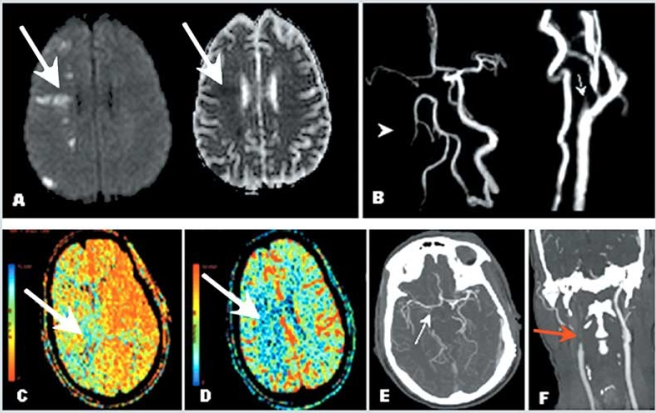
A 56-year-old right-handed man presented with confusion and left side weakness. He was found to have complete occlusion of the right internal carotid artery. Brain MRI demonstrated small infarctions in borderzone territories, located between anterior and middle cerebral artery, and between posterior and middle cerebral artery territories. Panel A demonstrates bright signal in the right hemisphere (Diffusion Weighted Imaging) and corresponding dark signal (Apparent Diffusion Coefficient, right of panel A) consistent with acute infarction. Magnetic Resonance Angiography (Panel B) and Computed Tomography Angiography of the neck and head (Panel F) confirmed occlusion of the proximal internal carotid artery after its origin (arrows). Panels B and E demonstrate lack of visualization of the right internal carotid artery intracranially as well as collateral flow through the anterior communicating artery suggested by visualization of the middle cerebral artery ipsilateral to the carotid occlusion. The patient presented severe worsening of left side weakness when anti hypertension medications were started, and developed left hemiplegia. A perfusion study (CTP) showed prolonged meant transit time (Panel C) and normal cerebral blood volume in the right middle cerebral artery territory (Panel D), consistent with a mismatch (ischemic penumbra). Repeat brain MRI showed no extension of the infarction (not shown). Collateral flow failure was suspected and blood pressure was elevated with intravenous fluids. The patient presented marked improvement and remained stable thereafter. He was discharged to rehabilitation, and at follow up 2 months later, had minimal left hemiparesis, was able to ambulate without supportive device and able to use fully his left hand. Modified Rankin Scale score was 1.

**Table 1:** Cerebral Collateral Circulation [15]

Intracranial		Arteries connected	Connecting artery
Circle of Willis		Internal carotid artery and Basilar / Posterior cerebral arteries	Posterior Communicating artery
		Anterior cerebral arteries	Anterior Communicating artery
Vertebrobasilar and circle of Willis		Internal carotid artery and vertebral / Basilar arteries	Trigeminal, Otic and Hypoglossal arteries
Tectal plexus		Posterior cerebral artery and superior cerebellar artery	Tectal rami, connecting supra and infratentorial arteries
Cerebral artery branches		Branches of the middle, anterior and posterior cerebral arteries	Anastomoses of terminal branches within and between arterial territories
Leptomeningeal	Pial plexus	Neighboring branches of major cerebral arteries	Arterioles from branches of same or adjacent arteries
	Meningeal	Cerebral and meningeal arteries	
**Extracranial**		**Arteries connected**	**Connecting artery**
Orbital plexus		Ophthalmic and Middle Meningeal, Maxillary, Ethmoidal arteries	Terminal branches
Rete mirabile caroticum		Internal and external carotid	

**Table 2:** Anatomic Variants of the Major Intracranial Arteries [14]

Artery	Variant	Frequency
Anterior cerebral (ACA)	Azygos ACA (unpaired ACA)	0-5%
	Medial ACA	3-22%
	Bihemispheric ACA	2-7%
	Persistent POA	Rare
Middle Cerebral	Duplicated	0.7-2.9%
Posterior cerebral (PCA)	Fetal PCA	20 – 30%
Anastomoses internal carotid-basilar arteries	Persistent trigeminal artery	0.1-1%
	Persistent hypoglossal artery	0.1-0.25%
	Proatlantal artery	Rare
